# Supplementary material for: Multi‐dataset identification of innovative feature genes and molecular mechanisms in keratoconus
Source: J Cell Mol Med. 2024 Sep 19;28(18):e70079. doi: 10.1111/jcmm.70079 (PMC11412914; doi:10.1111/jcmm.70079)
Supplement: Supplementary file 2 — Material S2. [file JCMM-28-e70079-s002.zip › 7.GSEA analysis/5.RNF217-AS1/KEGG_PENTOSE_AND_GLUCURONATE_INTERCONVERSIONS.html]

Details for gene set KEGG\_PENTOSE\_AND\_GLUCURONATE\_INTERCONVERSIONS[GSEA]

|  || Dataset | RNF217-AS1.group.cls#h\_versus\_l |
| Phenotype | group.cls#h\_versus\_l |
| Upregulated in class | h |
| GeneSet | KEGG\_PENTOSE\_AND\_GLUCURONATE\_INTERCONVERSIONS |
| Enrichment Score (ES) | 0.3970027 |
| Normalized Enrichment Score (NES) | 1.0577669 |
| Nominal p-value | 0.37572256 |
| FDR q-value | 1.0 |
| FWER p-Value | 0.998 |
Table: GSEA Results Summary

  

Fig 1: Enrichment plot: KEGG\_PENTOSE\_AND\_GLUCURONATE\_INTERCONVERSIONS      
 Profile of the Running ES Score & Positions of GeneSet Members on the Rank Ordered List

  

| SYMBOL | TITLE | RANK IN GENE LIST | RANK METRIC SCORE | RUNNING ES | CORE ENRICHMENT || 1 | UGT1A8 | na | 901 | 0.455 | 0.1665 | Yes |
| 2 | UGT2B4 | na | 3115 | 0.286 | 0.2238 | Yes |
| 3 | UGT1A6 | na | 4124 | 0.248 | 0.2998 | Yes |
| 4 | UGT2B11 | na | 5963 | 0.184 | 0.3249 | Yes |
| 5 | UGT2B28 | na | 7074 | 0.151 | 0.3568 | Yes |
| 6 | UGT2B10 | na | 9434 | 0.097 | 0.3297 | Yes |
| 7 | UGT2A3 | na | 10034 | 0.086 | 0.3488 | Yes |
| 8 | DHDH | na | 10141 | 0.084 | 0.3814 | Yes |
| 9 | RPEL1 | na | 10697 | 0.075 | 0.3970 | Yes |
| 10 | RPE | na | 13464 | 0.037 | 0.3329 | No |
| 11 | UGT2A1 | na | 14187 | 0.028 | 0.3241 | No |
| 12 | DCXR | na | 16536 | 0.003 | 0.2579 | No |
| 13 | UGDH | na | 18451 | -0.015 | 0.2091 | No |
| 14 | UGP2 | na | 19834 | -0.028 | 0.1812 | No |
| 15 | GUSB | na | 19854 | -0.029 | 0.1928 | No |
| 16 | CRYL1 | na | 23639 | -0.070 | 0.1135 | No |
| 17 | XYLB | na | 24973 | -0.090 | 0.1131 | No |
| 18 | UGT2B7 | na | 26756 | -0.121 | 0.1130 | No |
| 19 | UGT2B15 | na | 26916 | -0.124 | 0.1608 | No |
| 20 | AKR1B1 | na | 28116 | -0.152 | 0.1905 | No |
Table: GSEA details [plain text format]

  

Fig 2: KEGG\_PENTOSE\_AND\_GLUCURONATE\_INTERCONVERSIONS      
 Blue-Pink O' Gram in the Space of the Analyzed GeneSet

  

Fig 3: KEGG\_PENTOSE\_AND\_GLUCURONATE\_INTERCONVERSIONS: Random ES distribution      
 Gene set null distribution of ES for **KEGG\_PENTOSE\_AND\_GLUCURONATE\_INTERCONVERSIONS**
